# Editorial: Kidney cancer awareness month 2025: current progress and future prospects on kidney cancer prevention, diagnosis and treatment

**DOI:** 10.3389/fonc.2026.2018903

**Published:** 2026-10-20

**Authors:** Angelo Mottaran, John Peter Sfakianos, Abdallah Flaifel, Vitaly Margulis

**Affiliations:** 1 Division of Urology, IRCCS Azienda Ospedaliero-Universitaria di Bologna, Bologna, Italy; 2 University of Bologna, Bologna, Italy; 3 Icahn School of Medicine at Mount Sinai, New York, NY, United States; 4 New York University, New York, NY, United States; 5 University of Texas Southwestern Medical Center, Dallas, TX, United States

**Keywords:** biomarkers, immunotherapy, kidney cancer, molecular classification, rare renal tumors, renal cell carcinoma, kidney cancer diagnosis, kidney cancer treatment

## Introduction

March is Kidney Cancer Awareness Month, and this Research Topic was launched to mark it by gathering recent work on the prevention, diagnosis and treatment of kidney cancer. The burden is considerable: GLOBOCAN estimated about 434,000 new cases and 156,000 deaths worldwide in 2022 (1). Renal cell carcinoma (RCC) accounts for most of these tumors, and, as outlined in the call for this collection, more than 30% of patients with RCC are diagnosed with metastatic disease, which underscores the need for earlier detection and more effective therapy.

Progress over the past decade has been substantial. Combinations of immune checkpoint inhibitors, alone or with vascular endothelial growth factor receptor tyrosine kinase inhibitors, have reshaped the care of advanced disease, and the 2022 World Health Organization classification introduced molecularly defined categories of RCC (2). Progress in prevention and early detection has been slower, and the evidence guiding the care of rare tumors and unusual presentations remains thin.

The Research Topic closed with 23 published articles: nine Original Research articles, one Systematic Review, two Reviews and 11 Case Reports, contributed by teams from many countries. We group them into four themes: biology, risk and biomarkers; diagnosis; rare and aggressive histologies; and treatment (**Figure 1**).

**Figure 1:**
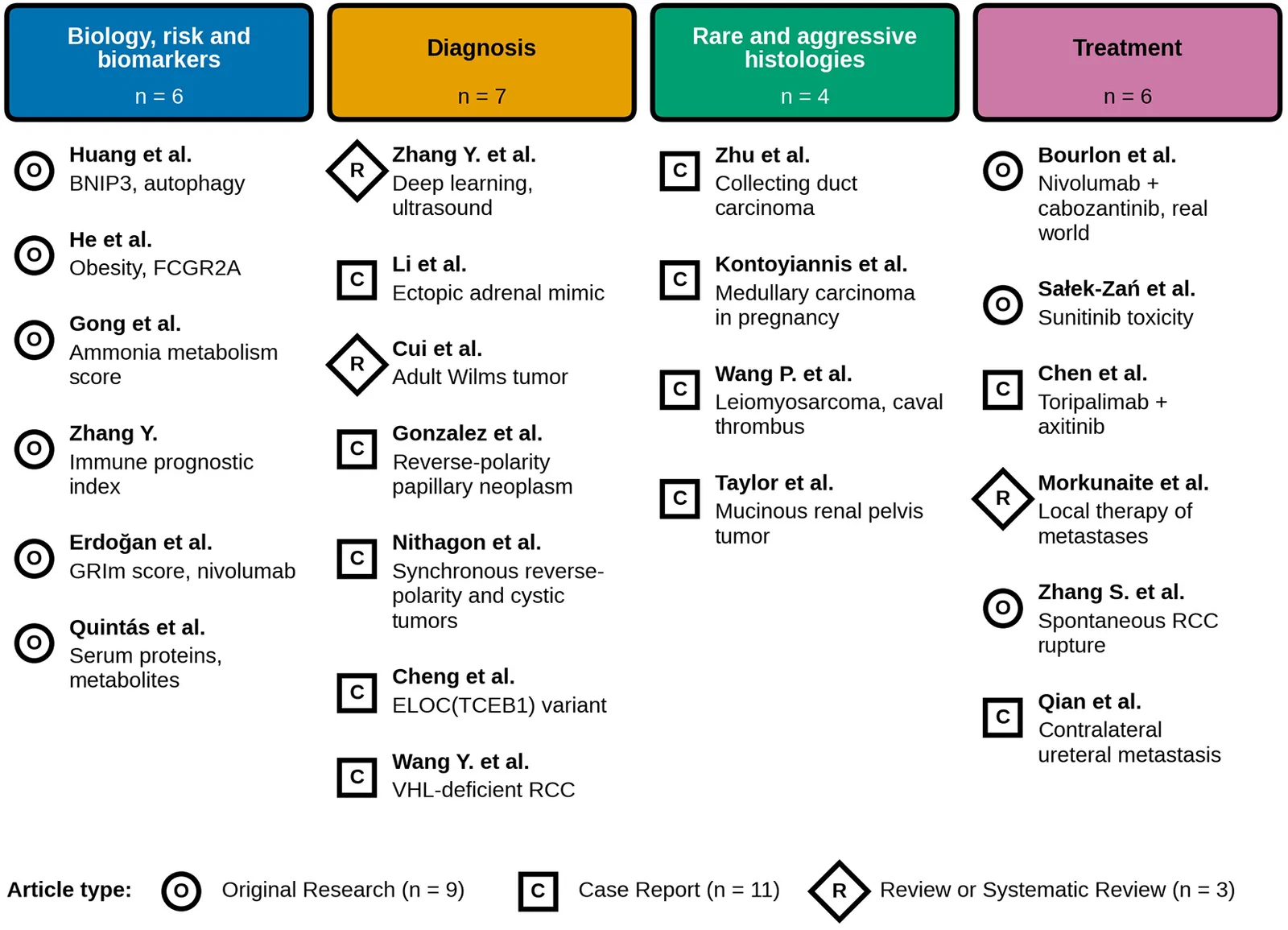
Overview of the 23 articles published in the Research Topic, grouped by theme and identified by first author. Symbols indicate article type: O, Original Research; C, Case Report; R, Review or Systematic Review. n, number of articles per theme; RCC, renal cell carcinoma.

## Biology, risk and biomarkers

Understanding why kidney tumors arise and how they behave is a precondition for prevention and for precision therapy. Huang et al. showed, in RCC cell lines and mouse xenografts, that overexpression of BNIP3, a hypoxia-regulated pro-apoptotic protein, reduced proliferation and invasion and increased apoptosis through hypoxia-inducible factor 1α–dependent autophagy, identifying autophagy as a potentially targetable vulnerability.

Obesity is an established risk modifier for clear cell RCC (ccRCC), yet how metabolic dysfunction shapes tumor biology is poorly defined. He et al. combined transcriptomic data from five ccRCC cohorts with obesity-related adipose profiles and machine learning, and identified *FCGR2A*, localized to M2-polarized macrophages in single-cell data, as a hub gene at the interface of the two conditions. Gong et al. similarly derived an ammonia metabolism–based risk score from public data, validated it in external and immunotherapy cohorts, and showed that silencing one of its genes, *CSAD*, reduced migration and invasion in four ccRCC cell lines. Both studies are hypothesis-generating, and both rest largely on retrospective transcriptomic datasets.

Blood-based and clinical markers are inexpensive and easy to transfer to practice. Zhang  proposed a Renal Immune Prognostic Index that combines prealbumin, lymphocyte count and immunoglobulin M in patients undergoing surgery, with validation in an independent cohort of 350 patients. Erdoğan et al. evaluated the Gustave Roussy Immune score (albumin, lactate dehydrogenase and neutrophil-to-lymphocyte ratio) in 81 patients treated with nivolumab in second or later lines, and found that low scores were associated with higher response rates and longer survival. The only prospective study in the collection, by Quintás et al. for the Spanish Oncology Genitourinary Group, measured serum proteins and metabolites in 38 patients receiving first-line sunitinib: higher interleukin-6, arginase-1 and S100A9 predicted shorter survival, and tryptophan–kynurenine metabolism differed between patients with extreme outcomes. These signatures now need prospective and external confirmation, ideally in patients treated with current first-line combinations.

## Diagnosis

Diagnosis begins with imaging. Zhang et al. reviewed deep learning across the renal ultrasound workflow, including segmentation, volumetry, functional prediction and disease diagnosis, and report performance approaching that of specialists for structure recognition and lesion detection; they identify data quality, interpretability, cross-device generalization and clinical integration as the main barriers. The review addresses kidney disease broadly rather than cancer alone, but it illustrates how widely available imaging could support earlier and more consistent detection.

Imaging can also mislead. Li et al. described a man with hematuria whose 1.6-cm enhancing renal-pole nodule was treated as RCC by partial nephrectomy and proved to be ectopic adrenal tissue, and Cui et al. reported an epithelial-type Wilms tumor, a malignancy typical of childhood, in a 23-year-old woman with a large renal mass. Both cases show that renal masses in adults can mimic RCC and that histopathology remains decisive.

Pathology and genomics increasingly define renal tumors. Gonzalez et al. and Nithagon et al. describe papillary renal neoplasm with reverse polarity, a recently characterized, *KRAS*-associated tumor with indolent behavior whose classification is still evolving. Nithagon et al. report what they describe as the first synchronous occurrence, in one kidney, of this tumor and a multilocular cystic renal neoplasm of low malignant potential, with genetically distinct profiles and no recurrence at nine months. Gonzalez et al. add PD-L1 expression in their case and note that only about 100 cases had been published. Cheng et al. reported a novel *TCEB1* variant (p.V73E) in an *ELOC(TCEB1)*-mutant RCC, confirmed by next-generation and Sanger sequencing, and Wang et al. presented two patients with VHL-deficient RCC managed with radiofrequency ablation, partial nephrectomy and/or pazopanib, discussing genetic testing and screening of relatives. Taken together, these reports show that accurate classification often requires immunohistochemistry and sequencing, and that it matters: indolent entities may be suited to nephron-sparing surgery, whereas hereditary syndromes call for family-level action.

## Rare and aggressive histologies

Several contributions concern tumors for which no standard exists. Zhu et al. reported collecting duct carcinoma, a rare, aggressive subtype in which most patients present with metastasis, in a 66-year-old man with hematuria; after nephrectomy he did not receive systemic therapy for financial reasons and was alive at 13 months, a reminder that access to treatment also shapes outcomes. Kontoyiannis et al. described what they report as the first documented renal medullary carcinoma diagnosed during pregnancy. Because this tumor is resistant to checkpoint inhibitors and antiangiogenic agents, chemotherapy with doxorubicin, cyclophosphamide and weekly paclitaxel was chosen for its safety profile in pregnancy; it produced tumor shrinkage, allowed delivery of a healthy infant at 33 weeks, and was followed by chemoradiation, and the patient was alive two years after diagnosis.

Wang et al. reported a renal leiomyosarcoma with inferior vena cava tumor thrombus whose caval disease progressed despite radical nephrectomy and adjuvant chemotherapy, in keeping with the poor prognosis of this entity. Finally, Taylor et al. characterized a mucinous adenocarcinoma of the renal pelvis, a tumor of the urinary collecting system rather than the renal parenchyma, arising in the setting of chronic stones and xanthogranulomatous pyelonephritis. Circulating tumor DNA and serum markers signaled recurrence when imaging was still nonspecific, and adjuvant chemotherapy was borrowed from colorectal cancer guidelines, since no renal-specific protocol exists.

## Treatment

Systemic therapy is the most mature area, and real-world data are now complementing pivotal trials. Bourlon et al. report the ARON-1 experience with first-line nivolumab plus cabozantinib in 333 patients across 52 centers in 17 countries, including patients with poor performance status, non-clear cell histology or brain metastases who are usually excluded from trials. Estimated two-year overall survival (75%) and objective response rate (58%) were broadly in line with the pivotal trial (3); grade 3 or higher toxicity was 37%, lower than in the trial, which the authors attribute in part to less systematic adverse event reporting. Poor performance status, sarcomatoid differentiation, bone or brain metastases and International Metastatic RCC Database Consortium poor-risk disease were adverse prognostic factors. The authors caution that less standardized response assessment in routine practice limits comparison of progression-free survival with trial data.

Sałek-Zań et al. analyzed 96 patients treated with first-line sunitinib at a single institution and documented that multi-organ toxicity was common, affecting 81% of patients. The authors report that cardiovascular toxicity, and dose reductions for cardiovascular, hematologic or gastrointestinal toxicity, were associated with longer survival, and they suggest that such toxicities may reflect treatment efficacy. These observations are hypothesis-generating: in retrospective analyses, associations between events occurring on treatment and survival can be shaped by how long patients remain on therapy, so prospective confirmation is needed before dose adjustment is linked to outcome. The authors also underline the need to balance toxicity and treatment continuity. Chen et al. reported a response to toripalimab plus axitinib in metastatic chromophobe RCC with sarcomatoid differentiation, which they present as the first such case; the response was observed after eight months, without grade 3 or higher adverse events. A single case cannot establish efficacy, but it is rare evidence for a scarce subgroup.

Local treatments and complex presentations complete the picture. Morkunaite et al. systematically reviewed 13 studies of non-systemic treatment for metastatic RCC and found that complete metastasectomy was associated with better overall and progression-free survival than incomplete or no metastasectomy, while stereotactic body radiotherapy offered palliation and durable disease control; a case of cryoablation illustrates the approach. As the evidence is observational and derives from selected patients, these findings identify candidates for prospective trials rather than a standard. Zhang et al. analyzed 37 cases of spontaneous RCC rupture treated over ten years at one center: five-year cancer-specific survival was 80.1% and progression-free survival 68.8%. Tumor stage, grade and size, perirenal fat invasion and coagulation abnormalities were associated with outcomes, whereas timing and type of surgery were not, in univariate analyses of a small cohort. Qian et al. reported clear cell RCC with synchronous contralateral ureteral metastasis, which they note has been described in fewer than ten patients. A staged approach, partial nephrectomy followed by nephroureterectomy, was used, the patient had no evidence of metastasis at 14 months, and the authors call for a multi-institutional registry of such rare presentations.

## Gaps and future prospects

Three gaps emerge. First, prevention and early detection are underrepresented. Apart from the obesity-related analysis and familial screening in VHL disease, none of the 23 articles examines modifiable risk, population screening or awareness interventions, although obesity, hypertension and smoking are established risk factors. Artificial intelligence–assisted imaging, inexpensive biomarkers and risk-adapted follow-up of incidentally detected masses deserve prospective study.

Second, the evidence base is fragile. Only one study is prospective; most others are retrospective, small or single-patient. Prognostic signatures require external and prospective validation, and real-world series such as ARON-1 and the sunitinib cohort lack systematic quality-of-life data, as their authors acknowledge.

Third, rare tumors need infrastructure rather than isolated reports. Registries, shared molecular profiling, molecular tumor boards and, where appropriate, circulating tumor DNA monitoring would help, as the pregnancy-associated, hereditary, mucinous and ureteral-metastasis cases illustrate. Access to therapy, highlighted by the collecting duct carcinoma case, belongs on the same agenda.

## Conclusion

Taken together, these 23 articles show a field moving toward finer classification, better risk stratification and more individualized treatment, while also exposing how much remains to be learned about prevention, early detection and rare disease. Kidney Cancer Awareness Month is a fitting occasion to renew that commitment, and we thank all authors, reviewers and editors who made this collection possible.

## References

[1] Bray F Laversanne M Sung H Ferlay J Siegel RL Soerjomataram I . Global cancer statistics 2022: GLOBOCAN estimates of incidence and mortality worldwide for 36 cancers in 185 countries. CA Cancer J Clin. (2024) 74:229–63. doi: 10.3322/caac.21834 38572751

[2] Moch H Amin MB Berney DM Compérat EM Gill AJ Hartmann A . The 2022 World Health Organization classification of tumours of the urinary system and male genital organs—part A: renal, penile, and testicular tumours. Eur Urol. (2022) 82:458–68. doi: 10.1016/j.eururo.2022.06.016 35853783

[3] Choueiri TK Powles T Burotto M Escudier B Bourlon MT Zurawski B . Nivolumab plus cabozantinib versus sunitinib for advanced renal-cell carcinoma. N Engl J Med. (2021) 384:829–41. doi: 10.1056/NEJMoa2026982 33657295 PMC8436591

